# Physical activity, obesity, and quality of life among rural Australian cancer survivors: a cross-sectional study

**DOI:** 10.1007/s00520-023-07691-w

**Published:** 2023-03-20

**Authors:** Michael J. Leach, Georgina Barber, Stephanie Monacella, Philip Jamieson, Thi Trinh, Ngan Vo, Ulla Schmidt, Anny Byrne, Eli Ristevski

**Affiliations:** 1 School of Rural Health, Monash University, Bendigo, VIC Australia; 2 West Gippsland Healthcare Group, Warragul, VIC Australia; 3 School of Rural Health, Monash University, Warragul, VIC Australia

**Keywords:** Quality of life, Exercise, Obesity, Rural, Cancer, Survivorship

## Abstract

**Purpose:**

We aimed to describe physical activity (PA), obesity, and quality of life (QoL) among rural Australian cancer survivors, assess whether total and item-specific QoL are associated with sufficient PA and obesity, and assess whether PA and obesity interact with respect to QoL.

**Methods:**

In a cross-sectional study, convenience sampling was used to recruit adult cancer survivors via a chemotherapy day unit and allied health professionals at a rural hospital in Baw Baw Shire, Australia. Exclusion criteria were acute malnutrition and end-of-life care. PA and QoL were measured using Godin-Shephard and 7-item Functional Assessment of Cancer Therapy (FACT-G7) questionnaires, respectively. Factors associated with total and item-specific QoL were assessed via linear and logistic regression, respectively.

**Results:**

Among 103 rural cancer survivors, the median age was 66 years, 35% were sufficiently physically active, and 41% presented with obesity. Mean/median total QoL scores were 17 on the FACT-G7 scale (0–28; higher scores indicate better QoL). Sufficient PA was associated with better QoL ($\widehat{\upbeta }$=2.29; 95% confidence interval [CI] = 0.26, 4.33) and more energy (odds ratio [OR] = 4.00, 95% CI = 1.48, 10.78) while obesity was associated with worse QoL ($\widehat{\upbeta }$=-2.09; 95% CI = -4.17, -0.01) and more pain (OR = 3.88, 95% CI = 1.29, 11.68). The PA-obesity interaction was non-significant (*p*-value = 0.83).

**Conclusions:**

This is the first known study conducted among rural survivors of any cancer to find sufficient PA and obesity are associated with better and worse QoL, respectively. PA, weight management, and QoL—including energy and pain—should be considered when targeting and tailoring supportive care interventions for rural cancer survivors.

## Introduction

Cancer survivors may experience a range of disease- and treatment-related effects, including pain, fatigue, insomnia, and peripheral neuropathy [1], as well as more chronic comorbidities than those without a cancer diagnosis [2]. As a consequence, cancer survivors are predisposed to relatively poor quality of life (QoL) [2]. Furthermore, a cancer survivor’s residential location may influence their quality and quantity of life [3–6]. Studies from the United States (US), Canada, Australia, New Zealand, and European countries report that non-metropolitan (henceforth termed ‘rural’) cancer survivors have worse QoL [3, 4, 7] and lower survival [6] than their metropolitan counterparts.

Factors related to QoL could be modified to improve the lives of cancer survivors, particularly in rural areas where patient outcomes and the level of unmet needs are known to be worse [3–5, 7]. Such potentially modifiable factors may include physical activity and obesity. In countries such as the US and Australia, people residing in rural areas are significantly more likely to experience obesity and to be insufficiently physically active than their metropolitan counterparts [8, 9]. Internationally, sufficient physical activity has been shown to be associated with better QoL among cancer survivors diagnosed with particular tumour types, including bladder [10], breast [11–13], endometrial [14], lung [15], non-Hodgkinson’s lymphoma [16], and ovarian [17] cancers. Conversely, obesity has been shown to be associated with worse QoL among survivors of any tumour type in a nationally representative US sample [18] as well as particular tumour types, including breast [11, 19, 20], colorectal [19], endometrial [14], lymphoma [21], melanoma [19], prostate [19], and uterine [19] cancers. All but one of these studies [13] were conducted in metropolitan, state/province-wide or nation-wide samples [3–5, 7, 10–12, 14–17, 19–21]. The cross-sectional study by Vallance et al. [13] reported a positive association between sufficient physical activity and better QoL in a sample of rural Canadian breast cancer survivors. No known studies have assessed the associations of physical activity and obesity with total QoL and/or specific QoL components among rural survivors of any, as opposed to a specific, tumour type.

As obesity is considered to be a lifestyle disease [22], obesity and physical activity may interact or confound one another in relation to their associations with cancer-related QoL. Three known cross-sectional studies set in the US [19], Canada [14] and Australia [11], respectively, reported that physical activity and obesity did not interact in relation to their associations with cancer-related QoL. As these three studies were conducted among state-wide or nation-wide samples, it is unclear if such an interaction exists for rural or metropolitan cancer survivors considered separately.

Given these gaps in the literature, we aimed to investigate physical activity, obesity, and QoL among rural Australian survivors of any cancer by answering four research questions: (1) What are the levels of sufficient physical activity, obesity, and QoL among rural Australian cancer survivors? (2) Is QoL associated with each of sufficient physical activity and obesity among rural Australian cancer survivors? (3) Which specific components of QoL, if any, are associated with each of sufficient physical activity and obesity among rural Australian cancer survivors? (4) Do physical activity and obesity interact in relation to QoL among rural Australian cancer survivors?

## Methods

### Setting

This study is set in Baw Baw Shire—a local government area located within West Gippsland in the state of Victoria, Australia. In 2021, Baw Baw Shire had an estimated resident population of 57,626 people and a median age of 41 years [23]—slightly older than the metropolitan population of Victoria [24]. Baw Baw Shire’s suburbs and postcodes are all classified as non-metropolitan by the Modified Monash Model (MMM), with the shire’s two most populous suburbs (Warragul and Drouin) having an MMM score of 4 (medium rural town) [25, 26]. Rates of potentially modifiable risk factors such as obesity, inadequate fruit intake, harmful alcohol use, smoking, and insufficient physical activity are higher in Baw Baw Shire than state- or nation-wide [27]. In Baw Baw Shire, chemotherapy is offered one day a week at the local hospital’s chemotherapy day unit (CDU), which has the capacity to treat 18 patients per week on average. On clinic days, this CDU is resourced by a visiting medical oncologist and a visiting haematologist. The nearest radiotherapy center is 54 km away by road.

### Design and sampling

A cross-sectional study was conducted using baseline (pre-intervention) data collected as part of a prospective cohort study investigating a nutrition and physical activity health coaching intervention: the I.CAN program [28]. Participants were primarily recruited via convenience sampling of cancer patients who, over the period August 2017-December 2021, attended the CDU at a rural hospital in Baw Baw Shire. The I.CAN program was offered to all cancer patients at the particular CDU as part of routine care, subject to pre-defined inclusion and exclusion criteria. The following inclusion criteria were applied: aged 18 years or over and diagnosed with any type and stage of cancer. The latter inclusion criterion aligns with the National Coalition for Cancer Survivorship’s broad definition of cancer survivorship: “from the time of diagnosis and for the balance of life.” [29] Those experiencing acute malnutrition or receiving end-of-life care were excluded from the present study due to urgent, complex needs. In addition to the recruitment of participants through the CDU, some eligible participants were recruited over the same period through referrals from allied health professionals practicing in the same hospital.

### Data collection

Quantitative data on participants’ characteristics were collected via paper-based forms prior to manual entry into a secure, password-protected Microsoft Access database (Microsoft Corp., Redmond, WA, USA).

### Measures

In the present study, I.CAN data on participants’ QoL, demographics, cancer type, treatment status, physical activity, and body mass index (BMI) were used.

The outcome of interest, QoL, was assessed using the 7-Item Functional Assessment of Cancer Therapy – General (FACT-G7) instrument Version 4 [30, 31], which has been shown to be valid and reliable in cancer populations. We chose the FACT-G7 over the broader FACT-G [32] because it is relatively brief and, thus, may help to reduce patient and clinician burden while increasing the rate of response to all items. The FACT-G7 instrument comprises the following seven items describing components of a cancer patient’s QoL over the past week:

1. I have a lack of energy
2. I have nausea
3. I am able to enjoy life
4. I have pain
5. I am sleeping well
6. I worry that my condition will get worse
7. I am content with the quality of my life right now [31].

For each item, respondents choose one response from a five-point Likert scale: ‘Not at all’ (scored 0), ‘A little bit’ (scored 1), ‘Somewhat’ (scored 2), ‘Quite a bit’ (scored 3), and ‘Very much’ (scored 4). In the present study, the total FACT-G7 score was computed in accordance with the developers’ scoring guidelines [31]. This gave a total FACT-G7 score in the range 0–28 (inclusive), where a higher number indicates better QoL. The total FACT-G7 score and item-specific FACT-G7 scores were treated as continuous variables. Additionally, each of the seven FACT-G7 items was assessed as a separate binary variable with categories of ‘more’ (scores of 3 or 4) and ‘less’ (scores of 0, 1 or 2). In order to obtain larger reference groups and, thus, more stable effect estimates in (binary) logistic regression, item-specific FACT-G7 scores were reversed as needed when creating binary outcome variables.

In terms of demographic and clinical variables of interest, age at baseline was treated as both a continuous variable and a polytomous variable with three categories: < 65, 65–74, and ≥ 75 years. Binary variables were created for gender (male or female), Aboriginal and/or Torres Strait Islander origin (yes or no), and country of birth (Australia or country other than Australia). Cancer type was dichotomised into categories of ‘breast’ and ‘not breast’ due, firstly, to the high proportion of participants with breast cancer and, secondly, to the fact that the only known rural study investigating the association between physical activity and cancer-related QoL was conducted among breast cancer survivors [13]. Given some relevant past studies were conducted among cancer survivors defined from the time of treatment completion rather than diagnosis (e.g. an Australian study of breast cancer survivors [11]), treatment status in our study was defined as a binary variable with the following categories: current or completed/ceased.

Regarding lifestyle factors, physical activity was measured using the Godin-Shephard Leisure-Time Physical Activity Questionnaire [33]—a validated instrument that is commonly used in oncology research [34]. This questionnaire includes four questions about the frequency of strenuous, moderate, and mild exercise during a typical week [33]. In the present study, participants’ physical activity scores were considered both as a continuous variable as well as a binary variable with the following categories: sufficiently active (score ≥ 14) and insufficiently active (score < 14) [35]. BMI in kg/m^2^ was calculated from participants’ height and weight. BMI was treated as a continuous variable, a polytomous variable with the World Health Organization’s (WHO’s) six nutritional status categories, and a binary obesity variable with WHO-defined categories of ‘yes’ (BMI ≥ 30.0 kg/m^2^) and ‘no’ (BMI < 30.0 kg/m^2^) [36].

### Statistical analyses

Polytomous and binary variables were described in terms of the frequency and percentage. The Shapiro–Wilk test was used to check whether continuous variables were approximately normally distributed. The distributions of the total FACT-G7 score, Godin-Shephard score, and BMI were further assessed via boxplots visualising the five-number summary (minimum, lower quartile, median, upper quartile, and maximum). The interquartile range (IQR) was calculated by subtracting the lower quartile from the upper quartile. The normally distributed total FACT-G7 score was summarised using the mean (standard deviation [SD]). As the remaining continuous variables were non-normally distributed, they were summarised using the median (IQR). As varying definitions of cancer survivorship exist [8, 11, 37], the difference in FACT-G7 scores between treatment status groups was assessed using the two-samples t-test while differences in sufficient physical activity and obesity between treatment status groups were assessed using Pearson’s chi-squared tests.

Associations between the primary outcome of interest, the total FACT-G7 score, and all categorical explanatory variables (except the polytomous BMI variable) were assessed via univariable and multivariable linear regression. The multivariable linear regression model contained all categorical variables except the polytomous BMI variable, giving seven independent/exposure variables in total. This permitted the assessment of the two independent variables of interest, sufficient physical activity and obesity, as well as the effects of potential confounding factors. Fitting the univariable and multivariable linear regression models involved calculating regression coefficients ($\widehat{\upbeta }$s) as well as corresponding 95% confidence intervals (CIs) and *p*-values. After fitting the multivariable linear regression model, the statistical significance of the interaction between sufficient physical activity and obesity was also tested in the multivariable setting. If this interaction term was not statistically significant, then it was excluded from the final multivariable model.

Associations between the secondary outcomes of interest—the binary, item-specific FACT-G7 variables—and each of obesity and sufficient physical activity were assessed via univariable and multivariable logistic regression. Due to limited numbers of outcome events, obesity and physical activity were only adjusted for one another and age group in the multivariable logistic regression models.

Across all analyses, a *p*-value less than 0.05 denoted a statistically significant result at the 5% level of significance. A complete-case approach to handling missing data was followed and all analyses were conducted in Stata v15.0 (StataCorp, College Station, TX, USA).

### Ethical considerations

Ethics approval was obtained from West Gippsland Healthcare Group Research Ethics Committee (ID: ICAN), Latrobe Regional Hospital Human Research Ethics Committee (ID: 2020-14) and Monash University Human Research Ethics Committee (ID: 11890). Prior to providing informed consent in writing, all participants were provided with written and verbal information about the I.CAN study.

## Results

### Study population

Of the 112 eligible adult cancer survivors, 103 (92%) had complete baseline data on all variables of interest and were included for analysis. The nine excluded participants were five individuals with missing FACT-G7 data and four individuals with missing BMI data. The demographic, medical, physical activity, and anthropometric characteristics of all included participants are described in Table 1. Continuous age in years was non-normally distributed (*p*-value < 0.05) with a negative skew (data not shown). The median (IQR) age of participants was 66 (19) years (Table 1).

**Table 1:** Rural cancer survivors’ demographic, medical, physical activity, and anthropometric characteristics in relation to quality of life (*N* = 103)

			FACT-G7 score	Univariable effect			Multivariable effect
Characteristic	*n*^†^	%^†^	Mean (SD)	$\widehat{\upbeta }$ (95% CI)	*p*-value	$\widehat{\upbeta }$ (95% CI)	*p*-value
Demographic							
Age in years (Median, IQR)	65.7	19.4	–	–	–	–	–
Age group							
< 65 years	49	47.6%	16.84 (4.33)	0	–	0	–
65–74 years	38	36.9%	16.39 (5.41)	–0.44 (–2.47, 1.59)	0.667	–0.01 (–2.05, 2.02)	0.990
≥ 75 years	16	15.5%	19.38 (4.19)	2.54 (–0.17, 5.25)	0.066	2.68 (–0.19, 5.55)	0.067
Gender							
Female	75	72.8%	17.32 (4.55)	0	–	0	–
Male	28	27.2%	16.39 (5.45)	–0.93 (–3.04, 1.18)	0.386	–0.98 (–3.41, 1.44)	0.424
Aboriginal and/or Torres Strait Islander origin							
No	103	100%	17.07 (4.80)	–	–	–	–
Yes	0	0%	–	–	–	–	–
Country of birth							
Australia	89	86.4%	17.09 (4.63)	0	–	0	-
Country other than Australia	14	13.6%	16.93 (5.99)	–0.16 (–2.91, 2.59)	0.908	–1.65 (–4.42, 1.11)	0.239
Medical							
Cancer type							
Other^‡^	55	53.4%	16.85 (5.00)	0	–	0	–
Breast	48	46.6%	17.31 (4.60)	0.46 (–1.43, 2.35)	0.631	1.08 (–1.09, 3.24)	0.329
Treatment status							
Current	52	50.5%	17.13 (5.03)	0	–	0	–
Completed/ceased	51	49.5%	17.00 (4.61)	–0.13 (–2.02, 1.75)	0.888	–1.11 (–2.97, 0.75)	0.240
Physical activity							
Sufficient physical activity^§^							
No	67	65.0%	16.04 (4.81)	0	–	0	–
Yes	36	35.0%	18.97 (4.21)	2.93 (1.04, 4.82)	0.003	2.29 (0.26, 4.33)	0.028
Anthropometry							
BMI group^¶^							
Underweight (BMI < 18.5 kg/m^2^)	2	1.9%	16.00 (4.24)	–	–	–	–
Normal weight (18.5 kg/m^2^ ≤ BMI < 25.0 kg/m^2^)	25	24.3%	17.68 (4.76)	–	–	–	–
Pre-obesity (25.0 kg/m^2^ ≤ BMI < 30.0 kg/m^2^)	34	33.0%	18.59 (5.18)	–	–	–	–
Obesity class I (30.0 kg/m^2^ ≤ BMI < 35.0 kg/m^2^)	25	24.3%	15.92 (4.06)	–	–	–	–
Obesity class II (35.0 kg/m^2^ ≤ BMI < 40.0 kg/m^2^)	11	10.7%	14.45 (4.99)	–	–	–	–
Obesity class III (40.0 kg/m^2^ ≤ BMI)	6	5.8%	15.83 (3.25)	–	–	–	–
Obesity^¶^							
No (BMI < 30 kg/m^2^)	61	59.2%	18.13 (4.94)	0	–	0	–
Yes (BMI ≥ 30 kg/m^2^)	42	40.8%	15.52 (4.17)	–2.61 (–4.46, -0.76)	0.006	–2.09 (–4.17, –0.01)	0.049

*n* frequency, $\widehat{\upbeta }$ estimated linear regression coefficient, *CI* confidence interval, *FACT-G7* Functional Assessment of Cancer Therapy – General (7-item version), *IQR* interquartile range, *BMI* body mass index, *kg* kilograms, *m* meters

^**†**^Unless otherwise stated

^‡^ ‘Other’ (i.e. non-breast) cancers include central nervous system, colorectal, genitourinary, gynaecological, haematological, lung, and upper gastrointestinal cancers

^§^Classified as a score ≥ 14 on the Godin-Shephard physical activity scale [35]

^¶^Classified using the World Health Organization’s definition [36]

### Level of quality of life

**Fig. 1:**
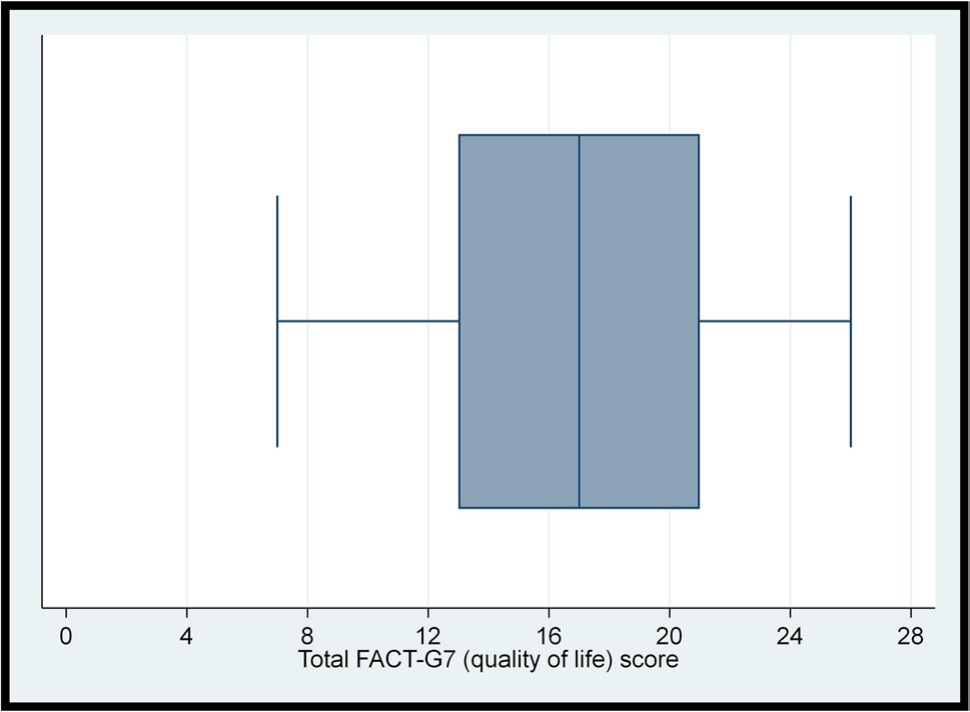
Boxplot for participants’ total FACT-G7 (quality of life) score (N = 103)

The total FACT-G7 score was approximately normally distributed (*p*-value = 0.20; Fig. 1). The mean (SD) total FACT-G7 score was 17.07 (4.80) (Table 2) on the scale of 0–28 while the corresponding median (IQR) score was 17.00 (8.00). Among the seven individual FACT-G7 items, the lowest mean score (1.68 on the scale of 0–4, SD = 1.09) was observed for ‘I *do not* have a lack of energy’ whereas the highest mean score (3.39 on the scale of 0–4, SD = 0.91) was observed for ‘I *do not* have nausea’ (Table 2).

**Table 2:** Descriptive statistics for individual FACT-G7 items and the total FACT-G7 score (N = 103)

FACT-G7 Items	Mean (SD)
1. I *do not* have a lack of energy^**†**^	1.68 (1.09)
2. I *do not* have nausea^**†**^	3.39 (0.91)
3. I am able to enjoy life^**†**^	2.74 (0.96)
4. I *do not* have pain^**†**^	2.52 (1.24)
5. I am sleeping well^**†**^	2.18 (1.35)
6. I *do not* worry that my condition will get worse^**†**^	2.29 (1.45)
7. I am content with the quality of my life right now^**†**^	2.26 (1.16)
Total FACT-G7 Score^‡^	17.07 (4.80)

*FACT-G7* Functional Assessment of Cancer Therapy-General (7-item version), *SD* standard deviation

^**†**^Item-specific score in the range 0–4, where 0 = Not at all, 1 = A little bit, 2 = Somewhat, 3 = Quite a bit, and 4 = Very much

^‡^Total score in the range 0–28. For each individual participant, this total score is the sum of the 7 item-specific scores

Table 1 shows the mean (SD) total FACT-G7 score for each category of participants’ demographic, medical, physical activity, and anthropometric characteristics. The mean (SD) FACT-G7 score did not significantly differ (*p*-value = 0.89) between the 51 participants who had completed/ceased treatment (17.00 [4.61]) and the 52 participant who were currently receiving treatment (17.13 [5.03]).

### Level of physical activity

**Fig. 2:**
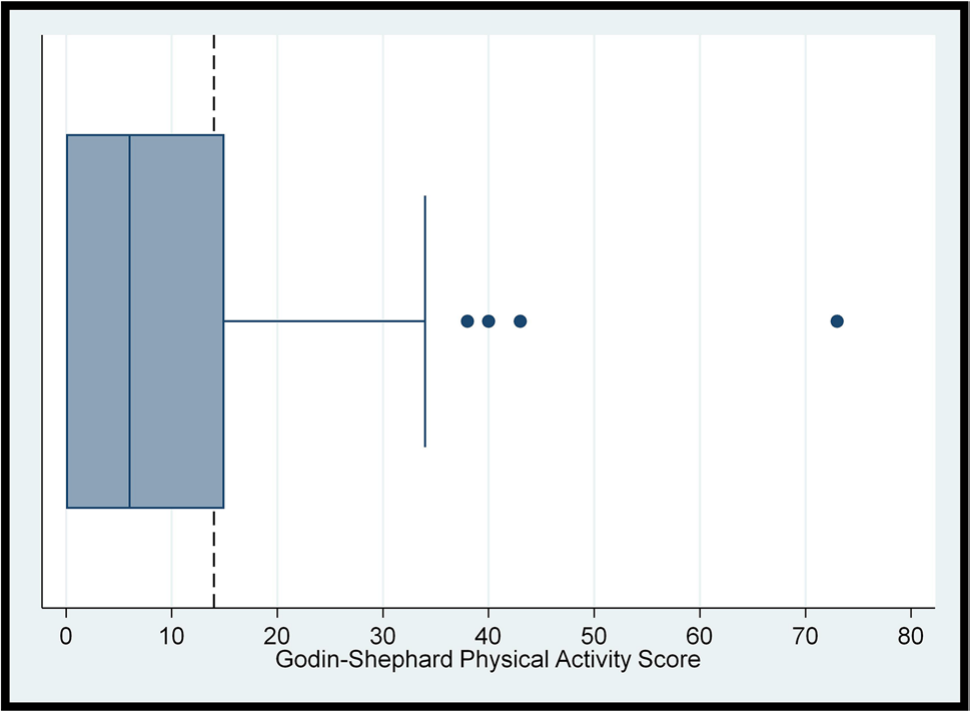
Boxplot for participants’ Godin-Shephard score (*N* = 103), with a dashed reference line at 14 denoting the threshold for sufficient physical activity

The Godin-Shephard physical activity score was non-normally distributed (p < 0.05) with a positive skew (Fig. 2). The median (IQR) Godin-Shephard physical activity score was 6.00 (15.00). Overall, 35% of participants had sufficient physical activity (Table 1). Regarding stratification by treatment status, 43% of the 51 participants who had completed/ceased treatment and 27% of the 52 participants currently receiving treatment were sufficiently physically active. This difference was not statistically significant (*p*-value = 0.08).

### Level of BMI

**Fig. 3:**
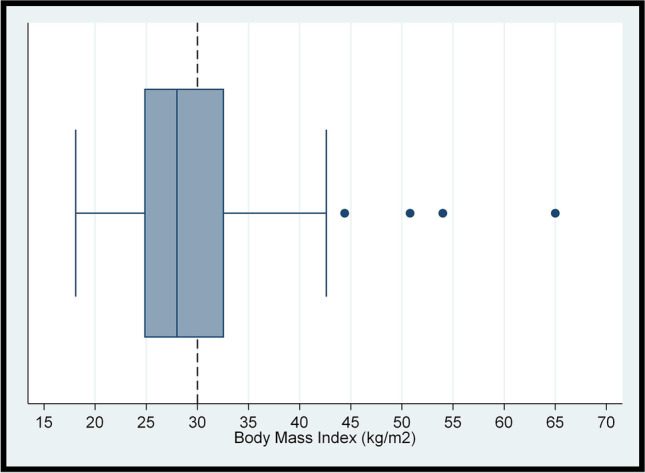
Boxplot for participants’ body mass index (*N* = 103), with a dashed reference line at 30 kg/m^2^ denoting the threshold for obesity

BMI was non-normally distributed (p < 0.05) with a positive skew (Fig. 3). The median (IQR) BMI was 28.00 (7.80) kg/m^2^. Forty-one per cent of the sample was classified into the obesity category (Table 1). Regarding stratification by treatment status, 37% of the 51 participants who had completed/ceased treatment and 44% of the 52 participants currently receiving treatment were people with obesity—a non-significant difference (p = 0.48).

### Correlates of QoL

Table 1 shows the univariable and multivariable linear regression results for factors associated with QoL. The two independent variables of primary interest—sufficient physical activity and obesity—were both found to be significantly associated with QoL. More specifically, independent of all other variables in the multivariable model, sufficient physical activity was associated with significantly better QoL while obesity was associated with significantly worse QoL. As the physical activity-obesity interaction was not statistically significant (*p*-value = 0.83), this interaction term was not included in the multivariable model. All other factors adjusted for in the multivariable model—age, gender, country of birth, cancer type, and treatment status—were not associated with QoL.

Due to small numbers of outcome events, only five of the seven item-specific FACT-7 scores could be collapsed into binary variables and used as outcomes in logistic regression models. The five binary outcome variables were ‘more energy’, ‘more pain’, ‘less worry’, ‘more sleep’ and ‘more content’. The adjusted odds of having more energy were significantly increased for those participants with sufficient physical activity, while the adjusted odds of experiencing more pain were significantly increased for those with obesity (Table 3).

**Table 3:** Associations between individual FACT-G7 items and each of sufficient physical activity and obesity (*N* = 103)

	aOR (95% CI)^†^				
Characteristic	More energy	More pain	Less worry	More sleep	More content
Sufficient physical activity^‡^					
No	1.00	1.00	1.00	1.00	1.00
Yes	4.00 (1.48–10.78)	0.82 (0.28–2.36)	0.98 (0.40–2.37)	2.18 (0.89–5.30)	1.84 (0.77–4.42)
Obesity^§^					
No (BMI < 30 kg/m^2^)	1.00	1.00	1.00	1.00	1.00
Yes (BMI ≥ 30 kg/m^2^)	0.67 (0.22–2.02)	3.88 (1.29–11.68)	0.78 (0.32–1.91)	1.28 (0.51–3.17)	0.67 (0.28–1.61)

*n* frequency, *aOR* adjusted odds ratio (in multivariable logistic regression model), *CI* confidence interval, *BMI* body mass index, *kg* kilograms, *m* meters

^**†**^In multivariable models, each of the variables ‘Sufficient physical activity’ and ‘Obesity’ were adjusted for one another as well as age

^‡^Classified as a score ≥ 14 on the Godin-Shephard physical activity scale [35]

^§^Classified using the World Health Organization’s definition [36]

## Discussion

Our study found the average level of total QoL in our sample of rural cancer survivors was 17 on the FACT-G7 scale—slightly higher than this instrument’s established cut-off value for low QoL (a score ≤ 16) [38]. While the average level of QoL in our sample was not low, the median FACT-G7 score was also 17. This means that nearly half of the sample would be considered to have low QoL. These low QoL scores were driven by the FACT-G7 item about lacking energy—a common problem that reportedly affects 10–27% of cancer survivors [39]. As no known studies set in rural areas have reported a mean or median FACT-G7 score, our study may provide a benchmark for any future rural studies conducted in samples with similar participant characteristics.

In our study, 41% of the sample presented with obesity while 35% had sufficient physical activity during a typical week. On the one hand, our study’s level of obesity is eight percentage points higher than the corresponding level of obesity in a study of rural South Australian cancer survivors: 33% [8]. On the other hand, the observed level of sufficient physical activity is five percentage points lower than the corresponding percentage of rural South Australian cancer survivors who had sufficient physical activity: 40% [8]. In terms of international comparisons, our study’s median BMI of 28 kg/m^2^ is similar to the mean BMI of 27 kg/m^2^ among rural Canadian breast cancer survivors [13]. However, the present study’s levels of sufficient physical activity post treatment (43%) and during treatment (27%) exceeded the corresponding percentages for rural Canadian breast cancer survivors (35% and 14%, respectively) [13].

Our study also found that sufficient physical activity was associated with significantly better QoL while obesity was associated with significantly worse QoL. The former result was driven by a significant association between sufficient physical activity and more energy while the latter result was driven by a significant association between obesity and more pain. The observed sufficient physical activity-QoL association aligns with past international studies conducted among survivors of particular tumour types [10–17], including a state-wide cross-sectional study of Western Australian breast cancer survivors [11] and a Canadian cross-sectional study of rural breast cancer survivors [13]. The obesity-QoL association in our sample is also supported in the broader literature [14, 18, 19, 21], as is the association between obesity and more pain [40]. The observed association between sufficient physical activity and more energy points towards a potential bidirectional association: those with less energy may feel less inclined to exercise while those who exercise may subsequently feel more energetic. This association could be investigated in future cohort studies. Ours is the first known study to find that total QoL and specific QoL items are associated with sufficient physical activity and obesity among rural cancer survivors. Pertinent supportive care interventions should be considered for rural cancer survivors experiencing physical inactivity, obesity and/or poor QoL, although any local barriers to supportive care screening and referrals among oncology staff (e.g. time constraints and scope of practice concerns [41]) would first need to be addressed.

The present study also found that, with respect to their associations with QoL, obesity did not interact with physical activity. This result aligns with those of past studies [11, 14, 19] while providing a novel finding for rural cancer survivors.

The results reported here should be interpreted in light of four key limitations. Firstly, as a cross-sectional study design was used, causation cannot be inferred from the statistically significant associations. For instance, it is unclear whether sufficient physical activity likely led to better QoL or better QoL likely led to sufficient physical activity. Secondly, unmeasured factors—most notably comorbid conditions [37]—could have confounded the observed associations between total/item-specific QoL and each of physical activity and obesity. Thirdly, the Godin-Shephard Leisure-Time Physical Activity Questionnaire [33] measures the frequency and intensity of physical activity but does not measure resistance activities, which are also recommended in clinical guidelines [42]. Fourthly, using a convenience approach to sampling eligible participants from one particular Australian setting has limited the generalisability of our findings.

Despite these limitations, key strengths of the present study include the use of validated measures of QoL [30] and physical activity [34], use of the standard international definition of obesity [36], and minimal missing data.

## Conclusion

In our sample of 103 rural Australian cancer survivors, approximately one-third were sufficiently physically active, 41% presented with obesity, and QoL on the FACT-G7 scale was, on average, 17—slightly higher than the cut-off of 16 for low QoL [38]. This is the first known study conducted in rural survivors of any cancer to find that sufficient physical activity and obesity are associated with better and worse QoL, respectively, and that these two factors do not interact with one another. This is also the first known study to find that, among rural cancer survivors, sufficient physical and obesity are associated with individual QoL items: more energy and more pain, respectively. Further studies are required to investigate these associations longitudinally among rural cancer survivors as well as their metropolitan counterparts. Nevertheless, this study’s findings suggest the need to improve physical activity, weight management, and QoL among rural cancer survivors. Supportive care interventions could be targeted and tailored to rural cancer survivors presenting with insufficient physical activity, obesity and/or poor QoL, including pain and low energy, potentially through supportive care screening and associated referrals to allied health professionals such as dietitians and exercise physiologists.

## Data Availability

The datasets that support the current study’s findings are not publicly available due to ethical/privacy restrictions. These datasets are, however, available from the corresponding author on reasonable request.
